# Phosphorylation Regulates the Bound Structure of an Intrinsically Disordered Protein: The p53-TAZ2 Case

**DOI:** 10.1371/journal.pone.0144284

**Published:** 2016-01-07

**Authors:** Raúl Esteban Ithuralde, Adrián Gustavo Turjanski

**Affiliations:** 1 Departamento de Química Inorgánica, Analítica y Química Física y Departamento de Química Biológica-Facultad de Ciencias Exacas y Naturales-Universidad de Buenos Aires, ciudad de Buenos Aires, Argentina; 2 INQUIMAE-Facultad de Ciencias Exactas y Naturales-Universidad de Buenos Aires/Consejo Nacional de Investigaciones Científicas y Técnicas-CONICET, ciudad de Buenos Aires, Argentina; Weizmann Institute of Science, ISRAEL

## Abstract

Disordered regions and Intrinsically Disordered Proteins (IDPs) are involved in critical cellular processes and may acquire a stable three-dimensional structure only upon binding to their partners. IDPs may follow a folding-after-binding process, known as induced folding, or a folding-before-binding process, known as conformational selection. The transcription factor p53 is involved in the regulation of cellular events that arise upon stress or DNA damage. The p53 domain structure is composed of an N-terminal transactivation domain (p53TAD), a DNA Binding Domain and a tetramerization domain. The activity of TAD is tightly regulated by interactions with cofactors, inhibitors and phosphorylation. To initiate transcription, p53TAD binds to the TAZ2 domain of CBP, a co-transcription factor, and undergoes a folding and binding process, as revealed by the recent NMR structure of the complex. The activity of p53 is regulated by phosphorylation at multiple sites on the TAD domain and recent studies have shown that modifications at three residues affect the binding towards TAZ2. However, we still do not know how these phosphorylations affect the structure of the bound state and, therefore, how they regulate the p53 function. In this work, we have used computational simulations to understand how phosphorylation affects the structure of the p53TAD:TAZ2 complex and regulates the recognition mechanism. Phosphorylation has been proposed to enhance binding by direct interaction with the folded protein or by changing the unbound conformation of IDPs, for example by pre-folding the protein favoring the recognition mechanism. Here, we show an interesting turn in the p53 case: phosphorylation mainly affects the bound structure of p53TAD, highlighting the complexity of IDP protein-protein interactions. Our results are in agreement with previous experimental studies, allowing a clear picture of how p53 is regulated by phosphorylation and giving new insights into how post-translational modifications can regulate the function of IDPs.

## Introduction

The classical paradigm states that the structure of a protein is related to its function. However, it has been shown that at least 30% of the human genome has no specific structure, including the whole gene or part of it, and that these regions generally bind to other proteins or DNA [1]. Disordered regions and Intrinsically Disordered Proteins (IDPs) are involved in critical cellular processes, such as the cell cycle. The deregulation of their function may lead to important health problems, such as Parkinson's disease, Alzheimer’s and cancer [1]. Disordered regions may acquire a stable three-dimensional structure upon binding to their partners, so the transition states of these processes are key to understanding this group of biomolecules [2–6]. In this sense there is great interest in unraveling the function of IDPs in cells. Experimental and computational studies show that many transcription factors exhibit large disordered regions that fold upon binding to their targets. Disordered regions may allow the protein to exhibit a greater capture radius, enhancing protein-protein recognition [1,7–8].

**Fig 1:**
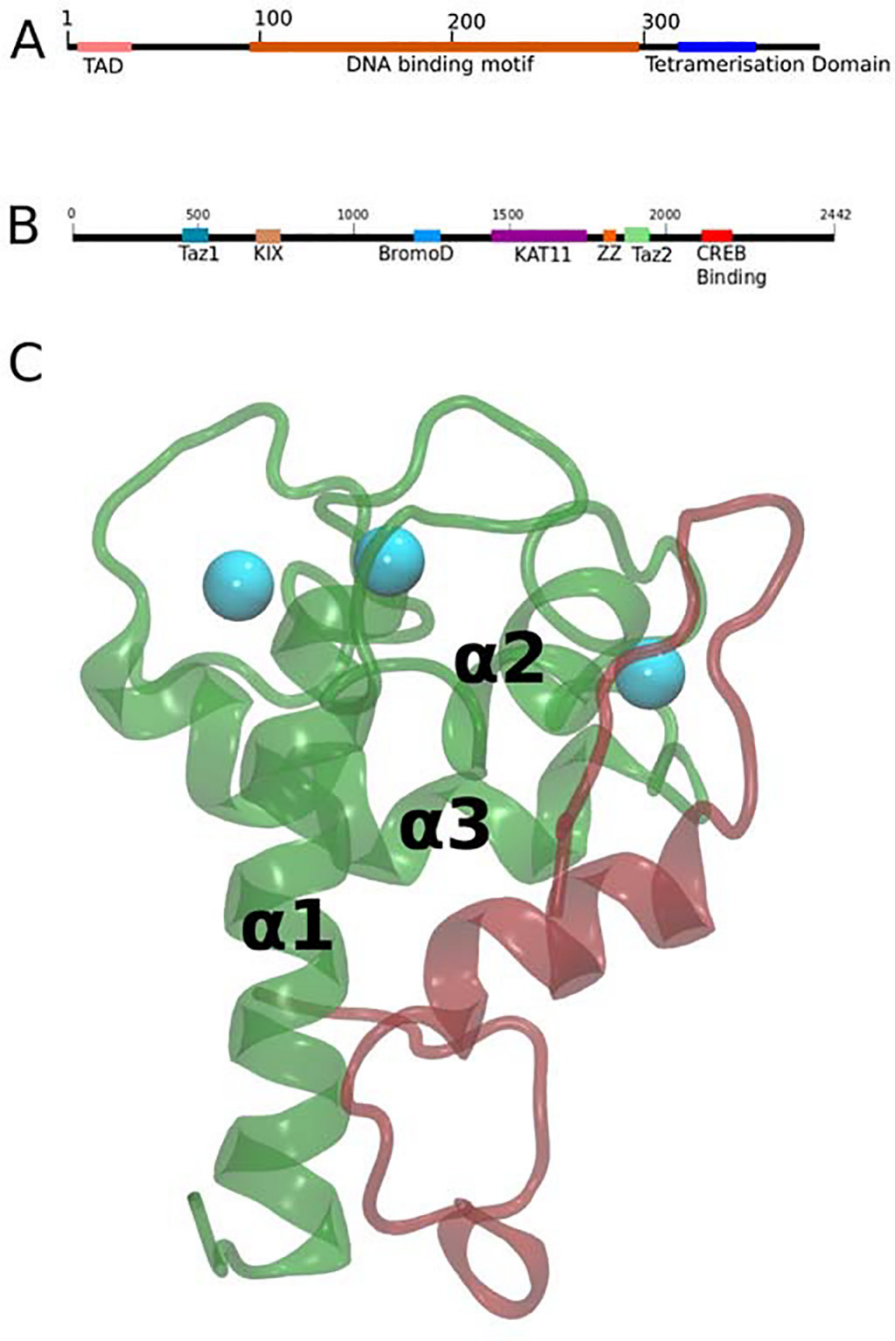
Structure of CBP and p53. (A) Domain structure of p53. (B) Domain structure of CREB Binding Protein (CBP). (C) The structure of the TAZ2 (green) domain of CBP with Zn^2+^ ions (cyan balls) bound to the p53TAD peptide (red). Ribbon representation is used for both domains.

p53 is a transcription factor involved in the regulation of cellular events that arise upon stress or DNA damage, such as apoptosis or cell cycle arrest. Its activity is tightly regulated by its interaction with cofactors and inhibitors and by post-translational modifications, such as ubiquitination, phosphorylation or acetylation [6,9–11]. The p53 domain structure is composed of an N-terminal transactivation domain (p53TAD), a DNA Binding Domain and a tetramerization domain (Fig 1). The p53TAD domain is intrinsically disordered but folds upon binding to other proteins, as the cofactor CREB Binding Protein (CBP), or the inhibitors MDM2 and MDMX [6].

In unstressed cells, it has been proposed that p53 is bound to the E3 ubiquitin-ligase MDM2 that catalyzes its ubiquitination and therefore regulates p53 degradation by proteasomes regulating p53 cellular levels [9–11]. Upon DNA damage, a series of cellular signaling cascades are activated, leading to p53 phosphorylation at multiple sites on the TAD. The p53TAD complex with MDM2 is mainly disrupted when Thr18 is phosphorylated, on the other hand it has been proposed that when p53 is phosphorylated at Ser15, Thr18 or Ser20 the affinity with the TAZ2 domain of CBP is increased. CBP is then able to bind and acetylate free p53, making it more stable [12].

CBP is a critical regulator of the transcription of numerous target genes, such as tyrosine hydroxylase, interleukin-6 and type I collagen, by interacting with specific transcription factors [13]. The domain structure of CBP is composed of TAZ1, KIX, Bromo, KAT11, ZZ, TAZ2 and CREB Binding (Fig 1). Tetramers of p53 can bind to the DNA, and the p53TAD of each monomer can bind to four distinct domains of CBP: TAZ1, TAZ2, KIX and IBiD. The TAZ2 domain is composed of three zinc-coordination sites and four alpha helices, which are named (from N-terminal to C-terminal) α1, α2, α3 and α4 (Fig 1B) [14,15].

The structure of the p53TAD:TAZ2 complex has been solved by NMR and biophysical experiments have been conducted to characterize the effect of p53TAD phosphorylation on TAZ2 recognition. In the wild-type unphosphorylated p53TAD:TAZ2 NMR complex residues 19–25 of p53TAD form an alpha-helix (Fig 1) indicating that a folding and binding mechanism is involved in the recognition mechanism. As we only have the structure of unphosphorylated p53-TAZ2 it has been difficult to explain how phosphorylation of p53TAD affects the recognition mechanism. NMR studies were done for two of the phosphor states but the structure of the complex could not be determined, in part due to overlap in the chemical shifts of the HSQC experiments [14]. Phosphorylation of p53TAD has been suggested to modulate the structure of the unbound p53 protein, specifically Thr18 phosphorylation has been postulated to decrease alpha-helix content in the unbound p53TAD, and phosphorylation of Ser20 has been postulated to increase it [9,16]. Moreover, it has been suggested that phosphorylation affects its long-range dynamical properties [17] and, therefore, the interaction with the TAZ2 domain of CBP [16,18]. The binding constants of phosphorylated p53 at the three residues have been measured by means of ITC and fluorescence experiments and several mutants have been tested for their effects in a previous study [14]. In particular as phosphorylation introduces a negative residue, Arg1731A, Arg1732A and Arg1737A, have been tested to explain its role in the bound complex [14]. Remarkably, the binding of p53TAD to TAZ2 is enhanced at the three phosphorylation sites [14].

Despite the fact that the p53TAD:TAZ2 complex has been well characterized, we do not have a clear picture of how phosphorylation at the three key residues modulates the binding mechanism. Taking into account the significance of protein phosphorylation in IDPs and the physiological relevance of the p53 protein, we decided to study at atomic resolution and by means of computer simulations how phosphorylation at Ser15, Thr18 and Ser20 modulates the structure of p53 in the bound state and therefore modifies the binding mechanism to the TAZ2 domain of CBP. We used Molecular Dynamics(MD) to simulate the p53TAD:TAZ2 complexes: unphosphorylated and phosphorylated at p53TAD Ser15, Thr18 or Ser20. By using the FOLDX program to generate mutants and MD we have been able to reproduce previous experimental results and obtain a model of how phosphorylation regulates p53 binding to TAZ2. We show that, due to its intrinsic flexibility, phosphorylation in p53TAD produces changes in the bound structure of p53TAD to TAZ2, thereby modifying specific interactions that are relevant for affinity and specificity. We postulate that for IDPs, post-translational modifications may be important not only for modifying the enthalpy of binding or inducing a conformational change in the unbound protein, as has been previously shown, but also for modulating the structure of the bound complex and thereby allowing it to form new and specific contacts that can only be understood if the bound phosphorylated structures are analyzed. Our study contributes to the understanding of how IDPs recognize their targets.

## Materials and Methods

The initial structure files were obtained from the structure of the complex of p53TAD (1–39) and TAZ2 (1723–1812) registered in the Protein Data Bank (PDB ID: 2K8F) [14]. We selected the first structure of the 10 models that were deposited in the PDB for the simulations. The zinc atoms were taken from the X-ray structure of TAZ2 registered in the Protein Data Bank (PDB ID: 1F81) [15].

Phosphate groups were added to the p53TAD:TAZ2 complex and topology and coordinate files were created using the Leap program of the AmberTools package [19]. Three structures were modeled, phosphorylated at Ser15, Thr18 and Ser20 of p53TAD.

The AMBER99SBildn force field was used [20,21], the zinc ions were modeled using the dummy atom approach [22,23], and the Stitch parameters were used for the phosphoserine and phosphothreonine residues [24]. Then, the topology and restart files were converted to the GROMACS files using the amb2gmx.pl program [25], and the rest of the simulations were done using the GROMACS version 4.5.3 package [26–30].

Explicit water molecules and counter ions were added using the TIP3P [31] model for water. Periodic boundary conditions were used [32,33]. The systems were equilibrated in the NVT ensemble by running 25 ps long MD simulations using the Berendsen thermostat [34], and then, the temperature was slowly raised to 300 K while running another 25 ps long simulation. Finally, the system was equilibrated at 300 K using the Berendsen barostat [35] set to 1 bar and running 100 ps long MD simulations. During these processes, the CA atoms were restrained using a harmonic potential with a 84 kJ/(mol.Å^2^) constant for the thermalization and a 42 kJ/(mol.Å^2^) constant for the density equilibration.

For each system, 230 ns long MD simulations were performed with no restraints and a 1.6 fs time step. Additionally, 100 ns long MD simulations were run for each system with different initial velocities that were selected using random numbers with the Boltzmann populations at 300 K. This second simulation was started from the 30 ns frame of the first one (when the dynamics seem to be equilibrated). We show in the supplementary information that the two set of simulations provide similar results. The temperature and pressure were kept constant by the v-rescale thermostat algorithm set at 300 K with a 0.1 ps coupling constant [36] and the Parrinello-Rahman barostat [37] set at 1.0 bar with a 0.1 ps time coupling constant, respectively. The LINCS algorithm [38] was used for constraining the bonds that contained an H atom, and electrostatic interactions were calculated by the Particle Mesh Ewald decomposition algorithm [32,33].

The Arg1737A mutant was modeled using a photo of the simulation of the p53TAD:TAZ2 complex phosphorylated at Ser15 where the Arg1737 of TAZ2 was not making contact with the phosphoserine residue. The FOLDX algorithm was used to generate the mutant.

For each state, we did a clustering analysis with a 2 Å tolerance. We performed mutational studies by using the FOLDX algorithm [39–42] for comparison to the experimental values. We mutated all of the residues in both TAZ2 and p53TAD to Ala.

The VMD program was used for visualization of the dynamics to create the images of the proteins and to calculate the g(r) plots [43]. Secondary structure analysis by residue was done using the DSSP program as implemented in GROMACS [44].

The AMBERTools package was used to calculate the RMSF and RMSD plots [45].

## Results

The results are organized as follows: initially, the stability of the simulations is presented. Then, the structural and dynamical changes between the unphosphorylated state and the three phosphorylated states are presented. In each case, detailed comparisons to previous NMR, mutational and biophysical experiments are presented. *In silico* mutational analysis was performed to understand the contributions of the different amino acids to the folding and binding process. Overall, our simulations are in agreement with previous experiments and allow a deeper understanding of how phosphorylation regulates the interaction of TAZ2 with p53TAD.

### Stability of the MD simulations

**Fig 2:**
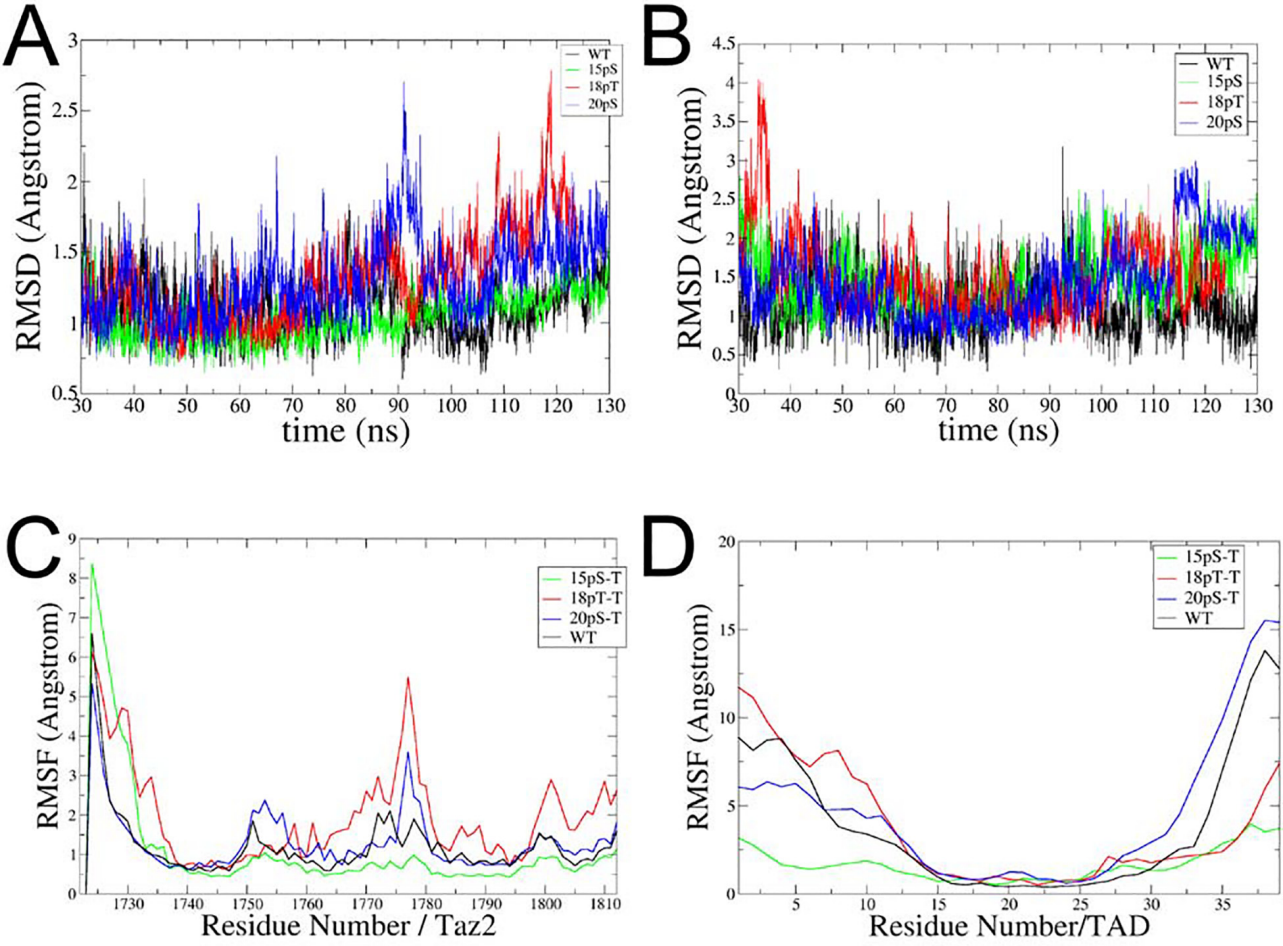
Backbone RMSD vs time and RMSF plots. (A) Plot of the Backbone RMSD of TAZ2 along the simulation. The MD was fitted against the average structure and only 30 ns-130 ns of the dynamics was used. (B) Backbone RMSD of p53TAD fitted against the average structure of the 30 ns-130 ns MD. (C) Backbone RMSF vs residue plot of TAZ2. The structure was fitted against the average structure of 30ns-130ns MD siulation. (D) Backbone RMSF vs residue plot of p53TAD. Again the structure was fitted against the average structure. In all cases p53 WT is black, p53 15pSer is green, p53 18pThr is red and p53 20pSer is blue.

We initially analyzed the stability of the simulations of the four systems studied, the p53TAD^WT^:TAZ2 complex and the same complex but with one phosphorylation at residues Ser15, Thr18 and Ser20 (p53 15pSer, 18pThr and 20pSer phosphorylated states respectively). In the case of the p53TAD peptide, as its tails are too flexible and have no definite secondary structure [14], we only calculated the Backbone RMSD for the p53TAD alpha helix. Fig 2 shows the Backbone RMSD vs time plot for the four systems, either for the whole TAZ2 domain or for the 14–27 segment of the p53TAD peptide. Similar results were obtained in another set of simulations as reported in the supplementary material (S2–S9 Figs).

The TAZ2 domain remains in a conformation similar to the NMR structure during the simulations, and despite its intrinsic flexibility, the overall fold remains stable. After the initial relaxation, encompassing the equilibration, and initial 30ns of MD, the four systems (WT, 15pSer, 18pThr and 20pSer) display moderate values for the RMSD of the 14–27 segment of the p53TAD peptide, using the average structure as a reference (Fig 2). Again, the two tails of the p53TAD peptides, WT and phosphorylated ones, are highly flexible and explore many different conformations within the simulations.

We also present a correlation of previously reported NMR chemical shifts for the CA for the complex and the ones calculated along the dynamics of the WT state with the SHIFTX program (S14 Fig). As can be seen there is a good agreement between simulations and experimental results for the native complex. We do not have the data of the NMR data of the phosphorylated complex, so we could not add a correlation figure in these cases.

We also analyzed the RMSF of the TAZ2 and p53 proteins (Fig 2C). In all of the simulations, there are no large differences in the TAZ2 domain in the four states. We did not observe large changes in the RMSF of p53TAD in the different states (Fig 2D) despite the fact that the 15pSer system reduces the fluctuation in both the N and C-terminal tails, which is likely due to a rotation of the central helices that accommodates the tail residues over the structure of the TAZ2 domain.

### Structural comparison of the p53TAD:TAZ2 complex in the different phosphorylation states

**Fig 3:**
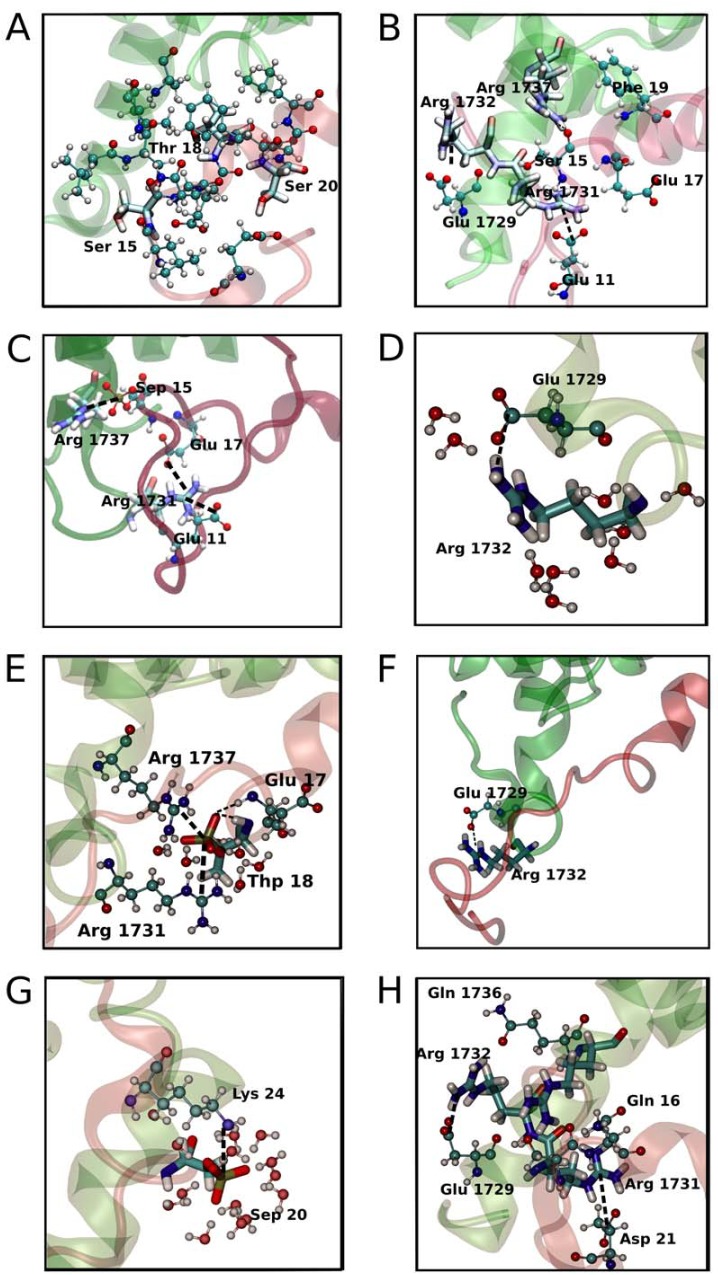
Changes in structure upon phosphorylation. (A) and (B) Structure of the Wild Type showing polar and charged inter protein interactions respectively. (C) and (D) depict the interactions of the15pSer and charged interactions respectively. (E) and (F) depict the interactions of the 18pThr and charged interactions respectively. (G and H) depict the interactions of the 20pSer and charged interactions respectively.

In this section we will discuss in detail the structural changes in the bound complex of the phosphorylation states. As the tails of the p53TAD peptide are highly flexible, in agreement with previous NMR experiments [14], and have weak interactions with the TAZ2 domain, will not be considered in the analysis. We separated the analysis in key residues previously reported to be relevant for the interaction of the two proteins. Key interactions are depicted in Fig 3.

#### Arginine residues in the α1 helix

Key interactions involve Arg and Lys residues of TAZ2 and phosphorylated and acidic residues of p53. In the WT protein Arg1731 interacts with Glu11, but we do not see any interaction with Glu17, which lies nearby (Fig 3B). Arg1731 interaction with Glu11 is observed in the 15pSer state but interaction Glu17 is observed but seems to be more transient (Fig 3C). In the 18pS state, the Arg1731 residue forms a salt bridge with the phosphate group and we also observed a salt bridge with Glu17 (Fig 3E). In the 20pSer state, Arg1731 interacts with p53TAD Asp21 (Fig 3H).

Arg1732 interacts in all states with the acidic residues of TAZ2, thus helping to maintain the secondary structure of the α1 helix (Fig 3).

Arg1737 is the most promiscuous of the three arginine residues. It interacts with Phe19 in the WT state (Fig 3B) but interacts with the phosphate group in the 15pSer and 18pThr (Fig 3C–3E) states and forms transient salt bridges with acidic N-terminal residues of p53TAD in the 20pSer state (Not shown). Interestingly, in our simulations the TAZ2 α1 helix bends around Arg1737 in the 18pThr state, which may explain the changes in chemical shifts of the amide resonances of Leu1733, Ile1735, Ala1738, Ile1739, Gln1740, Ser1741 and Leu1742 seen previously in the two dimensional 1H, 15N NMR HSQC spectra [14].

The interactions described for Arg1731, Arg1732 and Arg1737 may explain the relevance of these three positive residues in the binding process, as demonstrated by previous ITC experiments with TAZ2 mutants Arg1731Ala, Arg1732Ala and Arg1737Ala in the WT, 15pSer and 18pThr states, which show high and positive ΔΔG of binding [14] upon mutation. We will discuss the Arg1737Ala mutant in the 15pSer state in detail in the next section.

#### p53 Leu22 and Leu25

**Fig 4:**
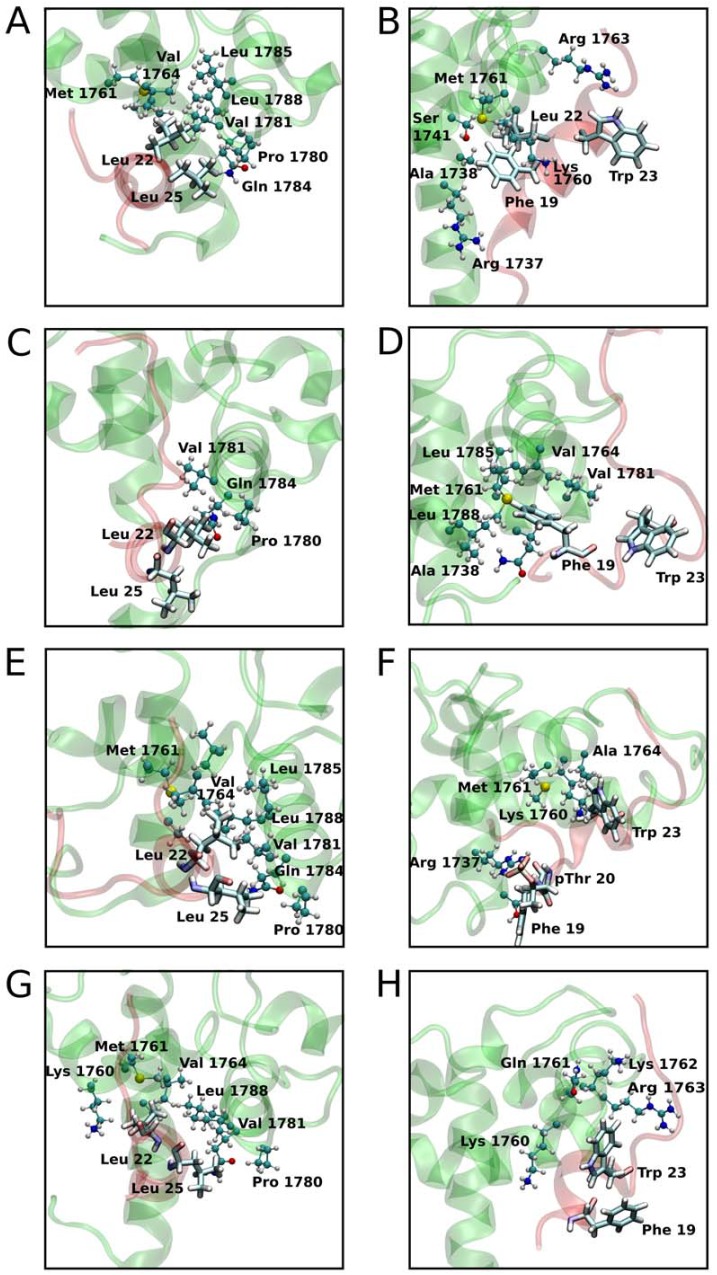
Changes in structure upon phosphorylation, showing interactions around the hydrophobic residues of p53TAD. (A) and (B) structures of the Wild Type protein showing aliphatic and aromatic residues of p53 interacting with TAZ2 respectively. (C) and (D) depict p53 15pSer-TAZ2 complex protein showing aliphatic and aromatic residues of p53 interacting with TAZ2 respectively. (E) and (F) depict p53 18pThr-TAZ2 protein complex showing aliphatic and aromatic residues of p53 interacting with TAZ2 respectively. (G) and (H) depict p53 20pSer-TAZ2 protein complex showing aliphatic and aromatic residues of p53 interacting with TAZ2 respectively.

In the WT state, Leu22 of p53 is inside a hydrophobic pocket formed by helix α1, α2 and α3 (Fig 4A) [14]. Leu25 interacts mainly with helix α3 of TAZ2. Leu22 and Leu25 also interact with each other (Fig 4A). Similar interactions are observed in all of the other states (Fig 4C, 4E and 4G). The mutational studies carried out with FoldX present positive ΔΔG of binding between TAZ2 and p53TAD for all states and results are presented in Table 1. The important intermolecular contacts observed during the simulations for Leu25 and Leu22 are in agreement with experimental data reported by Bai [14], which state that there is no binding between WT TAZ2 and p53TAD mutants Leu22Ala and Leu25Ala.

**Table 1:** ΔΔG of binding of TAZ2 and p53TAD for several mutations (kJ/mol).

Mutant	WT	15pSer	18pThr	20pSer
Arg1731Ala	1.53	3.87	1.34	1.41
Arg1732Ala	0.06	0.03	1.31	1.77
Arg1737Ala	0.12	1.52	0.74	1.00
Ser15Ala	-0.61	1.35	-1.08	-0.33
Thr18Ala	0.37	-0.34	0.56	0.07
Phe19Ala	2.38	3.69	2.16	1.03
Ser20Ala	0.04	-0.68	0.24	0.46
Leu22Ala	2.45	1.67	2.86	2.71
Trp23Ala	0.57	0.90	1.86	2.02
Leu25Ala	1.69	0.32	0.45	1.15

#### p53 Phe19

In the WT state, Phe19 is surrounded by the side chains of Arg1737 and Met1761 (Fig 4B). The aromatic residue also takes part in a cation pi interaction with Lys1760 [14]. Due to the rotation of the p53TAD helix, in the p53 15pSer state, the Phe19 side chain gets into a hydrophobic pocket formed by helix α1 and α2 and interacts with Leu1788 and Met1761 (Fig 4D). In the 18pThr state, Phe19 interacts with the hydrophobic regions of the α1 and α2 helix, as in the WT case, but due to the overall rotation of the peptide, it is now in a region more exposed to solvent (Fig 4F). In the 20pSer state, Phe19 is more exposed to the solvent (Fig 4H). In all states, we observe large and positive ΔΔG of binding between TAZ2 and Phe19Ala p53TAD with FoldX, as presented in Table 1. This is in agreement with experimental mutational studies for the WT state [14] that show decrease binding in the Phe19A mutants. The other aromatic residue of p53 Trp23 is less buried and maintains similar interactions in the different states as can be seen in Fig 4.

#### Phosphorylated residues

In the p53 15pSer state, TAZ2 residue Arg1737 is the main contact with the phosphate (Fig 3C).

We observed that the p53TAD pThr18 phosphate group interacts with Arg1731, Arg1737, and the amide backbone of Glu17. Interestingly, pThr18 also has a hydrogen bond with its own amide group (Fig 3E). We also note that the phosphate group is almost not exposed to the solvent, which again produces a rotation in the central helix that changes the secondary structure conformation of the p53TAD residues (S1 Fig).

The p53TAD pSer20 residue exhibits a phosphate group that interacts with its own Lys24 and is clearly more exposed to solvent (Fig 3G), as can be seen in the gofr plot (S1 Fig).

In all three cases, mutational studies using FoldX give large and positive ΔΔG of binding between TAZ2 and p53TAD, which is shown in Table 1. This results are in agreement with increase binding affinity of the phosphor proteins as compared to the WT [14]

#### The Arg1737Ala mutant of TAZ2 in complex with 15pSer

**Fig 5:**
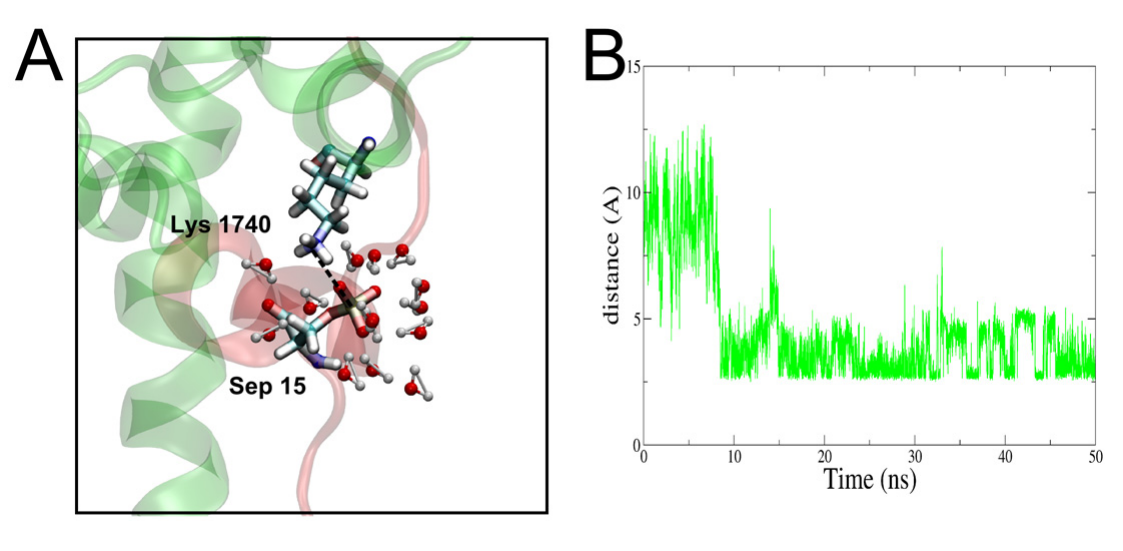
Dynamics and interactions in the Arg1737Ala mutant of p53 15pSer state. (A) Structure of Arg1737Ala mutant of p53 15pSer state, showing interactions between some residues around the phosphate group. (B) Distance of phosphorus atom of Sep15 and Nζ of Lys1740 versus time in the Arg1737Ala mutant of p53 15pSer state molecular dynamics.

A recent study with ITC experiments shows that there is no significant change in the Kd for the Arg1737Ala mutant in the 15pSer phosphorylation state, suggesting that it is not relevant for the complex [14]. However, we found that Arg1737 is the key residue interacting with the phosphate. We hypothesized that due to the overall high flexibility of the complex, once Arg1737 is mutated, other residues that had previously been exposed to solvent could replace the role of this positively charged residue in the interaction with the phosphoserine. To test this hypothesis, we performed molecular dynamics of the Arg1737Ala mutant based on our previous model. Interestingly, Lys1760 of TAZ2, which was previously exposed to solvent (ΔΔG of K1760A calculated by FoldX for the 15pSer molecular dynamics gives 0.92 kJ/mol, a relative low value), rotates and interacts with the phosphoserine, which could explain the observed experimental result and highlights the versatility of the protein complexes formed by intrinsically disordered proteins (Fig 5).

Table 2 summarizes the findings described in this section. We will now focus on the changes in the overall structure of p53 in the different states upon binding to CBP.

**Table 2:** Structural comparison between different phosphorylation states.

State	WT	p53 15pSer	p53 18pThr	p53 20pSer
Arg1731	Interacts with Glu11	Interacts with Glu11	Interacts with Thp18	Interacts with Asp21
Arg1732	Interacts with TAZ2 Asp1729	Interacts with TAZ2 Asp1729	Interacts with TAZ2 Asp1729	Interacts with TAZ2 Asp1729
Arg1737	Interacts with Phe19	Interacts with Sep15	Interacts with Thp18	Transient salt bridges with N-terminal acidic residues
p53 Leu22	Inside a hydrophobic pocket formed by helix α1, α2 and α3. Interacts with p53 Leu25.	Inside a hydrophobic pocket between helix α3 and α2. Interacts with p53 Leu25.	Inside a hydrophobic pocket between helix α3 and α2. Interacts with p53 Leu25.	Inside a hydrophobic pocket between helix α3 and α2. Interacts with p53 Phe19 and Leu25.
p53 Phe19	Arg1737 and Met1761.	Inside a hydrophobic pocket formed by helix α1 and α2	Inside a hydrophobic pocket formed by helix α1 and α2. Higher SASA than WT.	Exposed to solvent and interacts with p53 Leu22.
p53 phosphorylated residue		Interacts with Arg1737.	Interacts with Arg1731, Arg1737. H-bond with its own amide backbones and those of p53 Glu17.	Interacts with Lys24.
Extension of the p53 helix	15–22	16–21	16–20	19–22

### Secondary structure of the p53TAD 14–27 segment

Usually, the effect of phosphorylation of Intrinsically Disorder Proteins is analyzed in the unbound state. Previous work on an unbound p53TAD WT peptide in two different phosphorylation states suggests that phosphorylation at Thr18 destabilizes the helix, while phosphorylation at Ser20 stabilizes it [16]. These results can only be analyzed if we actually know the structure of the complex when p53 is phosphorylated. In a previous work, it is assumed to be very similar to the WT complex. The question now is whether the same results could be obtained in the complex with TAZ2, which would give insight into whether a pre-folded state is necessary for complex formation in the phosphorylated states.

**Fig 6:**
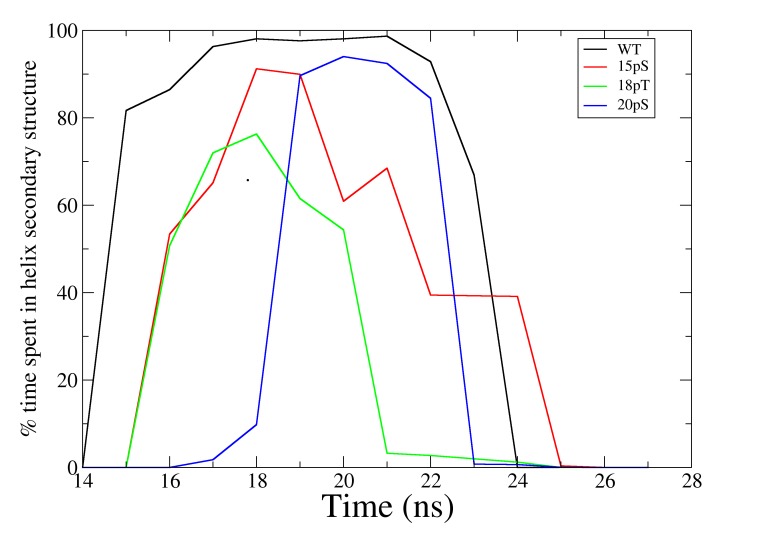
Percentage of time spent in a helix secondary structure versus number of p53TAD residues.

In the unphosphorylated complex a helix is observed, both in the NMR structure and during the MD simulations, around residues 18–26 and is stabilized by the interactions between Lys24 and Asp17 and 21. However, in all three phosphorylated states of the complex the helix rotates and becomes shorter with respect to the WT state. Moreover, the change in the secondary structure upon binding is less pronounced than in the WT state (Fig 6). A helix structure is formed between residues Asp21 and Leu25 in the 15pSer and 18pThr states (Fig 6). Interestingly, the interaction of Lys24 with other residues of the peptide is disrupted by the phosphate groups, which may partially destabilize the helix structure. In the 20pSer state, the helix is formed between residues 19–23. In the bound state, the central helix in the 18pThr and 20pSer states changes its position toward the N-terminal, around the phosphate group, and in the 15pSer has a kink at residues 20–21 that highly decreases the helical structure of p53. Similar results were obtained with another set of simulations as reported in supplementary material (S10, S11, S12 and S13 Figs).

The results presented in this section suggest that a pre-folded structure will not clearly favor the binding process, as the phosphorylated p53TAD peptides bound to TAZ2 show less tendency to form folded helix structures. Moreover, the reported tendency of the unbound phosphorylated peptides is not correlated with the helix formation in their bound state. PDB Files of all the structures are provided as supporting information (S1–S4 Files).

## Discussion and Conclusions

IDPs behave very differently than globular proteins, leading to a complex regulation of the mechanism of protein-protein recognition by changes in protein conformation.

Mutational studies have proven very useful in structural studies of globular proteins or protein domains; they are able to suggest important residues for the interaction between macromolecules and for stabilizing a secondary or tertiary structure needed for the interaction. However, as shown with the Arg1737 residue in the 15pSer state, in these highly flexible IDPs, low values of ΔΔG of binding does not always mean that the particular residue does not form part of the intermolecular interaction, as the mutated protein could replace the WT interaction by other ones, even when this imply changing the structure of a major part of the complex, as we have shown.

Two main mechanisms have been proposed for protein-protein interactions in IDPs: induced folding and conformational selection. The former mechanism implies that the IDP will bind disorderly and fold over the globular protein; in this sense, changes in the unbound state that stabilize a 'bound' conformation do not increase the binding rate. In the latter mechanism, changes that stabilize a bound-like conformation of the unbound IDP change the binding rate [46–48].

Possible changes in the unbound could be specific mutants designed to understand the binding mechanism or post-translational modifications, such as phosphorylation. It has been shown that many transcription factors are IDPs or have long intrinsically disordered regions and that phosphorylation is a common mechanism for regulating their function [1,6,7,49,50]. The role of phosphorylation could be to increase the equilibrium binding constant by specific interactions with co-transcription factors or by stabilizing the structure of the unbound protein and therefore changing the binding affinity by increasing the binding rate [14, 51–54]. The latter requires a conformational selection mechanism.

In the case of the p53TAD, different mechanisms have been proposed [14,16,18], and the role of phosphorylation has been studied both in its contribution to direct interactions with CBP and in producing changes of the structure of the unbound complex. By performing molecular dynamics simulations and *in silico* mutations, we have shown that phosphorylation probably regulates p53TAD by changing the structure of the bound complex and not only the unbound p53TAD. This changes can increase the binding constant by stabilizing contacts with p53TAD residues other than the phosphorylated one. According to our results, the cell may then be taking advantage of both phosphorylation and the plasticity of a disordered protein to modify the interaction of the protein complex by changing the bound structure. We believe this new path could be preferred in IDPs that do not undergo an important change in structure upon binding, and therefore, changes that affect the structure can clearly modulate the folding and binding process.

Our results not only help to understand p53 function but also contribute to the general knowledge of how IDPs recognize their targets.

## Supporting Information

S1 Fig gofr plot of the waters around the phosphate group. p53 15pSer state (green), p53 18pThr state (red) and p53 20pSer state (blue).
(TIFF)

S2 Fig Comparison of Backbone RMSD for first and second simulations. Backbone RMSD vs time plot for the first simulation (black), only 30 ns-130 ns dynamics were used, and the second simulation (red), all fitted against the TAZ2 backbone 30 ns photo for WT TAZ2.
(TIFF)

S3 Fig Comparison of Backbone RMSD for first and second simulations. Backbone RMSD vs time plot for the first simulation (black), only 30 ns-130 ns dynamics were used, and the second simulation (red), all fitted against the TAZ2 backbone 30 ns photo for WT TAD.
(TIFF)

S4 Fig Comparison of Backbone RMSD for first and second simulations. Backbone RMSD vs time plot for the first simulation (black), only 30 ns-130 ns dynamics were used, and the second simulation (red), all fitted against the TAZ2 backbone 30 ns photo for 15pSer-T TAZ2.
(EPS)

S5 Fig Comparison of Backbone RMSD for first and second simulations. Backbone RMSD vs time plot for the first simulation (black), only 30 ns-130 ns dynamics were used, and the second simulation (red), all fitted against the TAZ2 backbone 30 ns photo for 15pSer-T TAD.
(TIFF)

S6 Fig Comparison of Backbone RMSD for first and second simulations. Backbone RMSD vs time plot for the first simulation (black), only 30 ns-130 ns dynamics were used, and the second simulation (red), all fitted against the TAZ2 backbone 30 ns photo for 18pThr-T TAZ2.
(TIFF)

S7 Fig Comparison of Backbone RMSD for first and second simulations. Backbone RMSD vs time plot for the first simulation (black), only 30 ns-130 ns dynamics were used, and the second simulation (red), all fitted against the TAZ2 backbone 30 ns photo for 18pThr-T TAD.
(TIFF)

S8 Fig Comparison of Backbone RMSD for first and second simulations. Backbone RMSD vs time plot for the first simulation (black), only 30 ns-130 ns dynamics were used, and the second simulation (red), all fitted against the TAZ2 backbone 30 ns photo for 20pSer-T TAZ2.
(TIFF)

S9 Fig Comparison of Backbone RMSD for first and second simulations. Backbone RMSD vs time plot for the first simulation (black), only 30 ns-130 ns dynamics were used, and the second simulation (red), all fitted against the TAZ2 backbone 30 ns photo for 20pSer-T TAD.
(TIFF)

S10 Fig Comparison of TAD helix structure for first and second simulations. Percentage time spent in helix structure vs TAD Residue Number plot for the first simulation (black), only 30 ns-130 ns dynamics were used, and the second simulation (red) for the WT state.
(TIFF)

S11 Fig Comparison of TAD helix structure for first and second simulations. Percentage time spent in helix structure vs TAD Residue Number plot for the first simulation (black), only 30 ns-130 ns dynamics were used, and the second simulation (red) for the 15pSer-T state.
(TIFF)

S12 Fig Comparison of TAD helix structure for first and second simulations. Percentage time spent in helix structure vs TAD Residue Number plot for the first simulation (black), only 30 ns-130 ns dynamics were used, and the second simulation (red) for the 18pThr-T state.
(TIFF)

S13 Fig Comparison of TAD helix structure for first and second simulations. Percentage time spent in helix structure vs TAD Residue Number plot for the first simulation (black), only 30 ns-130 ns dynamics were used, and the second simulation (red) for the 20pSer-T.
(TIFF)

S14 Fig Agreement between simulated NMR CA chemical shifts and experimental CA chemical shifts for the WT complex. (TIFF)

S1 File Structure file of the WT state obtained from MD simulations. (PDB)

S2 File Structure file of the 15pSer state obtained from MD simulations. (PDB)

S3 File Structure file of the 18pThr state obtained from MD simulations. (PDB)

S4 File Structure file of the 20pSer state obtained from MD simulations. (PDB)
