# Supplementary figures and images for: Phosphorylation Regulates the Bound Structure of an Intrinsically Disordered Protein: The p53-TAZ2 Case
Source: PLoS One. 2016 Jan 7;11(1):e0144284. doi: 10.1371/journal.pone.0144284 (PMC4712144; doi:10.1371/journal.pone.0144284)

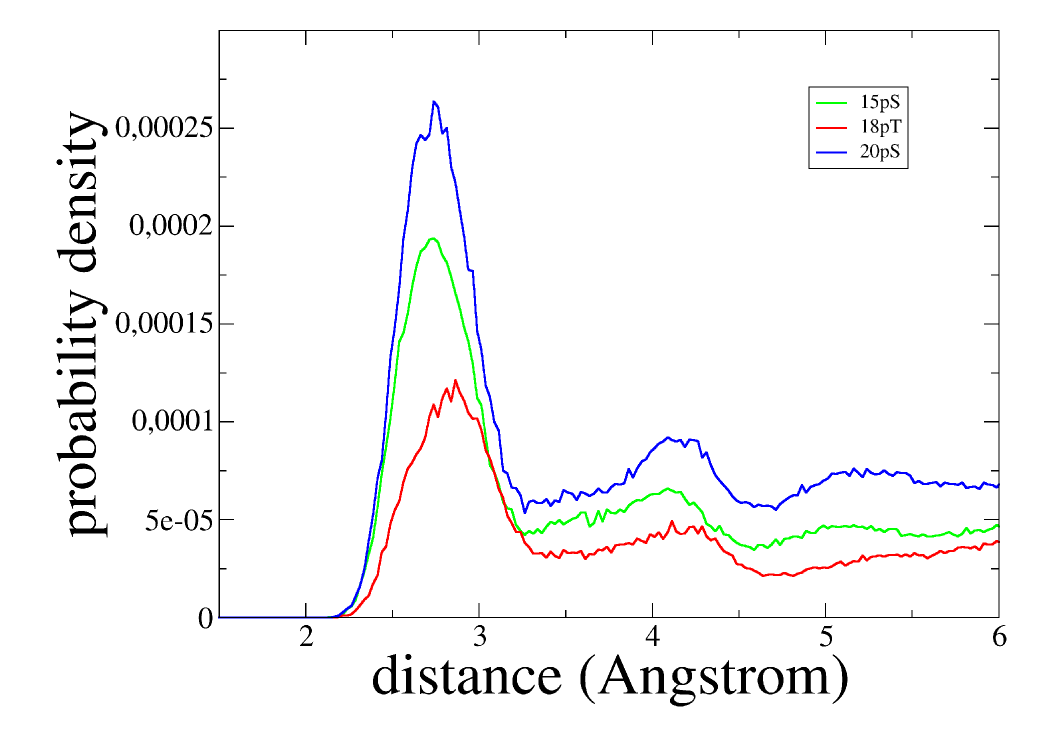

Supplement: S1 Fig — p53 15pSer state (green), p53 18pThr state (red) and p53 20pSer state (blue). (TIFF) [file pone.0144284.s001.tiff]

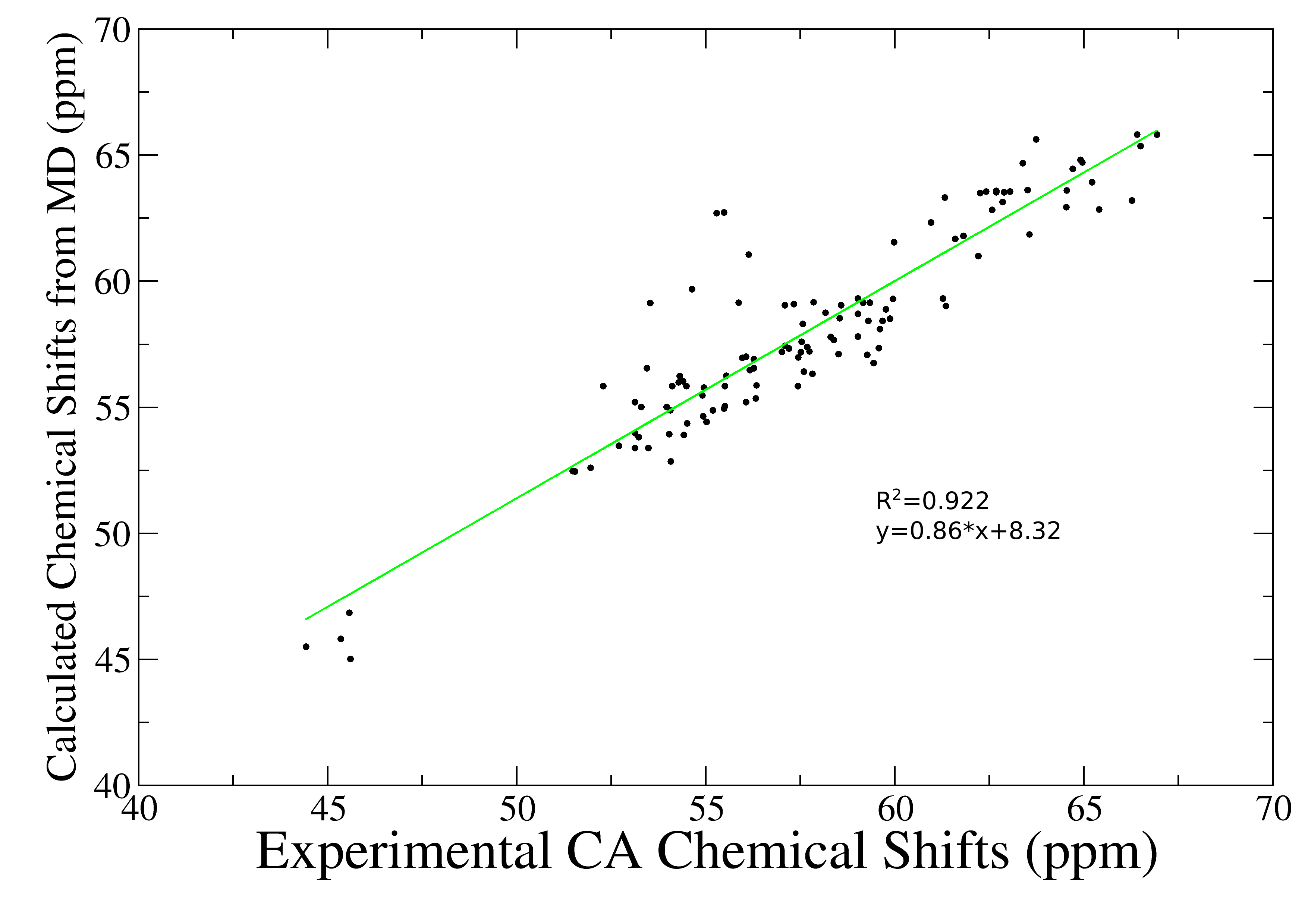

Supplement: S14 Fig — (TIFF) [file pone.0144284.s014.tiff]
